# Challenges in the Evolving Role of Calreticulin as a Promising Target for Precision Medicine in Myeloproliferative Neoplasms

**DOI:** 10.3390/cancers17213397

**Published:** 2025-10-22

**Authors:** Alessandro Costa, Massimo Breccia

**Affiliations:** 1 Hematology Unit, Businco Hospital, Department of Medical Sciences and Public Health, University of Cagliari, 09121 Cagliari, Italy; 2 Hematology, Department of Translational and Precision Medicine, Az. Policlinico Umberto I-Sapienza University, 00161 Rome, Italy

**Keywords:** myeloproliferative neoplasm, immunotherapy, target therapy, calreticulin, immune escape, monoclonal antibody, bispecific antibody

## Abstract

More than a decade after its discovery, advances have been made in understanding the oncogenic role of mutant *CALR* in *BCR::ABL1*-negative myeloproliferative neoplasms (MPNs). Disease biology has proven to be distinct from other MPN subtypes, with meaningful differences that have created opportunities for therapeutic targeting of *CALR*-mutant clones. Among the approaches under investigation, immunotherapy has advanced furthest into clinical development and holds promise. Several strategies are now being explored, including monoclonal antibodies directed against the CALR neoepitope, T-cell–redirecting bispecific antibodies, precision antibody–drug conjugates, vaccination approaches, and CAR T-cell therapies. Early-phase clinical trials with fully human anti-CALR monoclonal antibodies (e.g., INCA033989) have shown very promising hematologic and molecular responses with manageable toxicity. In preclinical models, bispecific antibodies and CAR T-cell therapy offer additional avenues to exploit the selective cell-surface localization of mutant CALR. By contrast, vaccination strategies have so far demonstrated limited clinical efficacy, and their potential in clinical practice remains challenging. At the same time, the complexity of *CALR*-driven disease raises key questions, including whether anti-CALR therapies can shift treatment goals beyond thrombotic risk reduction, how best to monitor clonal burden, and how to address immune escape. In this review, we highlight the latest therapeutic advances in *CALR*-mutated MPNs while outlining the critical unmet needs that will shape the future of care for these patients.

## Simple Summary

Calreticulin (CALR) mutations in myeloproliferative neoplasms (MPNs) create unique opportunities for targeted therapy. The most advanced approach involves monoclonal antibodies specifically designed to recognize the CALR neoepitope, with early clinical trials showing encouraging results. Additional preclinical data derives from bispecific antibodies that redirect T cells against *CALR*-mutant cells, precision antibody–drug conjugates delivering cytotoxic payloads, and CAR T-cell therapies. Vaccination against CALR-derived peptides has shown immune activity but limited benefit, and its role remains uncertain. This review discusses these advances and outlines the challenges that must be overcome to translate them into routine clinical practice.

## 1. Introduction

*BCR::ABL1*-negative myeloproliferative neoplasms (MPNs), including polycythemia vera (PV), essential thrombocythemia (ET), and primary myelofibrosis (PMF), are clonal hematopoietic stem cell disorders characterized by sustained myeloid proliferation and a variable risk of thrombosis, hemorrhage, and fibrotic or leukemic progression [1]. Their clonal origin was first suggested in the 1970s [2], but major advances in understanding occurred with the identification in 2005 of the *JAK2* V617F mutation, present in nearly all PV cases and in a majority of ET and PMF. Soon after, activating mutations in MPL, the thrombopoietin (TPO) receptor, were identified in a smaller subset of ET and PMF [3]. A further breakthrough came in 2013 with the identification of somatic insertions and deletions in exon 9 of the *CALR* gene, which encodes calreticulin, occurring in 20–25% of ET and PMF patients without *JAK2* or *MPL* mutations [4,5].

In recent years, interest in *CALR* mutation has steadily grown, shifting from their initial diagnostic and prognostic relevance to their potential as direct therapeutic targets. The unique biochemical features of mutant *CALR*, including its interaction with MPL and its immunogenic neoepitope, have opened new avenues for targeted therapies. Considering these advances, we examine the evolving significance of *CALR* in MPNs together with open issues for integrating immune-based strategies into the management of *CALR*-positive diseases.

## 2. Genetic and Molecular Pathways in CALR-Mutated MPNs

### 2.1. Structure and Function of Calreticulin

**Figure 1:**
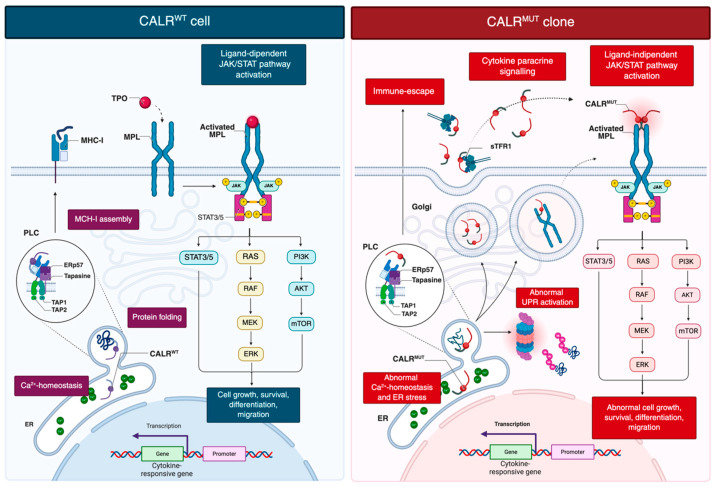
Current model of oncogenic signaling in *CALR*-mutant clones. CALR^wt^ contributes to protein folding, Ca^2+^ homeostasis in ER, and PLC-mediated MHC-I assembly. TPO-dependent activation of MPL drives ligand-mediated JAK/STAT signaling that support proliferation, survival, differentiation and migration. CALR^wt^ assists proper MPL folding while remaining ER-retained. CALR^mut^ lacks the C-terminal KDEL retention signal, enabling its dislocation into the Golgi and secretory pathway. The mutant C-terminus, generated by a frameshift, induces conformational changes in the N-domain, increasing accessibility to the lectin-binding pocket and facilitating aberrant interactions with immature N-glycans and the D1 domain of MPL. This interaction occurs in the ER and Golgi and allows the CALR^mut^-MPL complex to traffic to the cell surface, where CALR^mut^ acts as a cytokine, inducing ligand-independent MPL dimerization and constitutive JAK/STAT activation. Additional consequences include abnormal UPR activation, disrupted Ca^2+^ homeostasis, and immune escape due to defective MHC-I assembly. Created in BioRender. Costa, A. (2025) https://BioRender.com/6cdrn75. Abbreviations: CALR, calreticulin; ER, endoplasmic reticulum; MHC-I, major histocompatibility complex class I; MPN, myeloproliferative neoplasm; PLC, peptide-loading complex; sTFR1, soluble transferrin receptor 1; TPO, thrombopoietin; UPR, unfolded protein response.

Wild-type CALR is a conserved endoplasmic reticulum (ER) lumen chaperone essential for intracellular calcium (Ca^2+^) homeostasis, protein quality control, and glycoprotein folding (Figure 1) [6].

The *CALR* gene comprises nine exons and encodes a protein organized into three functional domains: an N-terminal lectin-like domain that binds glycans, a central proline-rich (P) domain that interacts with polypeptides, and a C-terminal domain ending with the ER-retention signal KDEL (Lys-Asp-Glu-Leu) [7].

Functionally, the C-terminal domain binds Ca^2+^ with low affinity, thereby contributing to ER calcium buffering [8]. The KDEL sequence ensures retention of CALR within the ER, where it facilitates the proper folding of N-glycosylated proteins destined for the plasma membrane, extracellular space, or other cellular compartments [9]. As a holdase chaperone, CALR preferentially binds glycosylated precursors through its lectin domain, preventing aggregation, premature oligomerization, oxidation, and degradation of misfolded proteins [10].

CALR also plays a key role as an extracellular “eat-me” signal expressed on the surface of stressed or apoptotic cells. Once externalized, CALR binds to phosphatidylserine and functions as a damage-associated molecular pattern (DAMP), thereby promoting macrophage-mediated phagocytosis [11]. The P domain of CALR has been proposed to serve as a molecular bridge to phagocytic receptors, including low-density lipoprotein receptor-related protein 1 (LRP1) and C1q, enhancing its pro-phagocytic function [12]. In parallel, activated macrophages have been shown to secrete CALR, which binds to viable target cells and marks them for clearance through a mechanism of programmed cell removal (PrCR) [13].

### 2.2. Advances in Molecular Mechanisms and Oncogenic Transformation of CALR-Mutated MPNs

Since their discovery in 2013, more than fifty distinct *CALR* mutations have been identified. All are located within exon 9 and produce a +1 base pair (bp) frameshift [5]. Based on structural features and the extent of acidic domain loss, they are categorized as type 1 mutations (52-bp deletion; CALR^Del52^), with complete loss of the acidic C-terminal region, or type 2 mutations (5-bp insertion; CALR^Ins5^), with partial retention of the acidic domain [4,5]. The remaining 20% are designated as type 1-like or type 2-like mostly according to structural predictions based on α-helical content. Mutants with higher predicted α-helical content are considered type 2-like, whereas those with lower content resemble type 1 [14,15,16].

Structural studies have provided insight into the abnormal interaction between mutant CALR and MPL. Neither the intrinsic chaperone activity of CALR nor its polypeptide-binding domain is necessary for MPL activation. Instead, three features are critical: specific N-glycosylation sites on MPL, the lectin-binding ability of mutant CALR, and the positively charged residues in its novel C-terminal tail generated by the frameshift [17,18,19,20]. Replacing these basic residues with neutral glycine, or partially deleting the mutant tail, reduces the CALR transforming capacity without affecting MPL binding, indicating that receptor binding and activation are distinct processes [21].

Mutations in *CALR* have shown to affect key aspects of cellular biology and immune regulation (Figure 1) [22,23,24]. Loss of the ER-retention KDEL sequence directs mutant CALR into the secretory pathway, enabling pathological MPL activation [4,5]. The altered C-terminal tail triggers an N-domain conformational change, exposing the N-glycan binding pocket for recognition of immature N-glycans on MPL [25]. Direct electrostatic interactions are also thought to occur between the positively charged residues in the mutant C-terminal domain and negatively charged residues within the D1 domain of MPL [25]. This interaction, occurring in the ER and Golgi, facilitates trafficking of the CALR–MPL complex to the cell surface, where mutant CALR acts as a cytokine mimic, promoting ligand-independent MPL dimerization and sustained JAK/STAT activation [19,26]. Additional data indicate that homomultimerization of mutant CALR, driven by its aberrant C-terminal sequence, is necessary for full receptor activation [25,27]. Mutant CALR was also detected in plasma of MPN patients [28]. Pecquet et al. [28] investigated its biological role and demonstrated a correlation between soluble mutant CALR (smCALR) levels and mutational burden. They further showed that the prolonged half-life of smCALR results from its complex formation with soluble transferrin receptor 1 (sTFR1). Functionally, smCALR probably binds to and activates MPL on megakaryocyte progenitor cells, promoting ligand-independent proliferation and sensitizing neighboring cells to CALR-mediated signaling.

### 2.3. Clonal Dynamics and Open Challenges

Over the past decade, preclinical models and genomic analyses have provided critical insights into clonal evolution, tracing the trajectory of hematopoietic stem cells (HSCs) from early subclinical expansion to overt disease [29]. In particular, investigations into self-renewal dynamics and somatic mutation–based lineage tracing have shown that *JAK2* V617F mutations may arise decades before diagnosis [30], and in some cases even in utero [31]. A striking parallel has been reported for *CALR* in a unique case involving monozygotic twins who developed *CALR*-mutated MF in adulthood. Both individuals harbored the same somatic *CALR* mutation, likely acquired in one twin and transmitted to the other through transplacental hematopoietic cell sharing. Subsequent clonal divergence was evidenced by the presence of a *TET2* mutation in only one twin [32]. Mathematical modeling studies suggest that, unlike *JAK2*, *CALR* mutations may arise later in life but confers a stronger proliferative advantage, thereby accelerating clonal expansion [33]. Interestingly, some evidence showed that the proportion of mutated cells is not equally distributed within the progenitors, with *CALR*-mutated HSCs biased towards myeloid differentiation and *CALR*-mutated cells heavily enriched in megakaryocyte progenitors [23,34].

However, translational research on *CALR* remains limited by several constraints inherent to current models, including species-specific differences, reliance on ectopic overexpression, and limited phenotypic penetrance [35]. Transgenic mouse models, including Calr^Del52^;Calr+/+ and heterozygous knock-in lines, typically develop thrombocytosis without progression to fibrosis [36,37]. Homozygous models exhibit more pronounced features, such as marked thrombocytosis, reduced bone marrow erythropoiesis, splenomegaly, and extramedullary hematopoiesis, but lack a competitive repopulating advantage in transplantation assays [36,38,39]. More recently, it has also been shown that HSCs with *CALR* haploinsufficiency display MPN-like features, including reduced bone marrow erythropoiesis and splenic extramedullary hematopoiesis; the combination of CALR^Del52^ and CALR haploinsufficiency restores the self-renewal capacity of mutant HSCs, eliminating the competition from wild-type cells and aiding the expansion of mutant clones towards MPN [40].

Recently, CRISPR/Cas9 editing has enabled the targeted introduction of *CALR* mutations directly into the endogenous mouse locus, generating in-frame deletions of 19, 52, or 61 bp. However, these models also exhibit only mild thrombocytosis and limited JAK/STAT activation [41,42]. To overcome these limitations, a novel humanized knock-in model was developed in 2023, in which common *CALR* mutations were introduced into healthy human HSCs using CRISPR/Cas9 [35]. Consistent with earlier findings, *CALR*-mutated HSCs did not show immediate proliferative advantage, suggesting that additional transcriptional or microenvironmental events are required to drive transformation. Interestingly, some mice transplanted with human *CALR*-mutated HSCs developed marrow fibrosis and splenomegaly, potentially reflecting a greater sensitivity of human MPL to TPO compared with its murine counterpart [35]. These findings support the concept that *CALR*-driven transformation is not cell-autonomous but instead depends on a complex interplay between the driver mutation, intrinsic transcriptional programs, and extrinsic environmental signals. Nonetheless, further analyses are needed to fully understand the oncogenic dynamics of *CALR* and to overcome the limitations of current models.

## 3. Immunotherapeutic Strategies in CALR-Mutated MPNs

### 3.1. Anti-CALR Monoclonal Antibodies

The advent of immunotherapy has transformed the treatment landscape of hematologic malignancies. Among these, monoclonal antibodies (MoAbs) occupy a central role due to their ability to selectively target malignant cells, limiting off-target toxicity and enabling durable responses even in relapsed or refractory disease [43]. While well established in lymphoproliferative disorders and acute leukemias, their application in MPNs is more recent, yet they represent the most advanced immunotherapeutic approach in both preclinical and early clinical development (Table 1).

The cell-surface localization of mutant CALR makes it an attractive target. The first preclinical evidence, reported in 2020, described B3, a murine chimeric MoAb recognizing the mutant CALR C-terminal domain [44]. In *CALR*^Del52^ ET murine model, B3 reduced bone marrow megakaryocyte counts and improved thrombocytosis. Subsequently, Achyutuni et al. [38] treated homozygous *CALR*^Del52^ transgenic mice with a murine IgG2a antibody directed against the human CALR neoantigen, achieving a rapid decline in platelet counts after five doses over 2.5 days, with rebound to baseline within 24 h after discontinuation.

In 2022, two independent groups advanced this concept. Mughal et al. [45] generated antibodies targeting internal peptides within the mutant CALR C-terminal, defining key epitope. In parallel, Tvorogov et al. [46] developed 4D7, an IgG2α MoAb specific for a C-terminal sequence common to type 1 and type 2 CALR mutations. 4D7 selectively disrupted the mutant CALR-MPL interaction, attenuated hyperactivation of the JAK/STAT pathway, suppressed thrombopoietin-independent megakaryocytic proliferation, and inhibited differentiation of *CALR*-mutated CD34+ cells, prolonging survival in xenograft models, including ruxolitinib-resistant disease.

**Figure 2:**
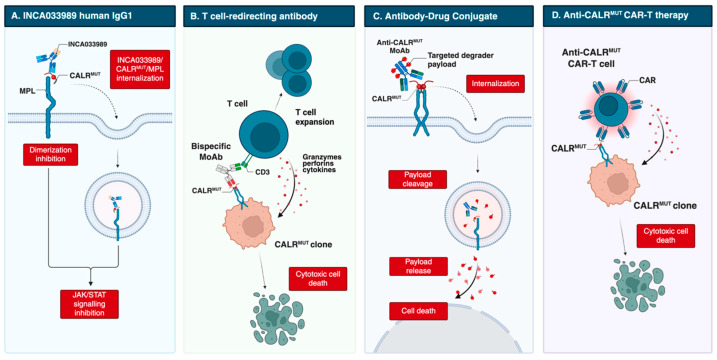
Emerging therapeutic strategies targeting mutant CALR, including direct MoAb, T-cell redirecting bispecific Ab, antibody-drug conjugate and anti-CALR CAR-T cell therapy. Created in BioRender. Costa, A. (2025) https://BioRender.com/vwehboc. Abbreviations: CAR-T, chimeric antigen receptor T cell; MoAb, monoclonal antibody.

However, these antibodies were generated in non-human species and had incompletely characterized mechanisms of action. A major advance came with INCA033989, developed by Reis et al. [47]. This fully human IgG1 MoAb harbors an N297A Fc mutation that abolishes Fc-mediated effector functions (Figure 2). INCA033989 binds recombinant CALR^Del52^ (K_d_ 1.75 nM) and CALR^Ins5^ (K_d_ 6.78 nM), with preferential binding to CALR^Del52^, likely reflecting epitope exposure differences in the CALR^Del52^-MPL complex. The MoAb/CALR/MPL complex relies on dynamin-dependent endocytosis; whether mutant CALR or antibody binding affects MPL recycling remains unclear. Functionally, INCA033989 inhibited pSTAT3/pSTAT5 in *CALR*-mutated MF CD34+ cells in a dose-dependent manner, without affecting wild-type, *JAK2*-mutated, or *MPL*-mutated cells. In a *CALR*-mutated ET murine model, it induced hematologic and molecular responses by selectively targeting mutant hematopoietic stem cells while sparing normal hematopoiesis. In a *CALR*^Del52^/*TP53* Q317 double-mutant post-MPN AML xenograft, it selectively targeted mutant cells and prolonged survival [48].

To date, two phase 1 clinical trials are underway for INCA033989, specifically the INCA33989-101 (NCT05936359, ex-US) and INCA3989-192 (NCT06034002, US). Both are multicenter, open-label, first-in-human studies with a phase 1a dose-escalation stage in high-risk MF or ET patients harboring exon 9 *CALR* mutations, followed by phase 1b expansion and phase 1c crossover with ruxolitinib in MF patients with suboptimal response. Preliminary data in high-risk ET (*n* = 49) treated with doses ranging from 24 mg to 2500 mg administered intravenously every two weeks show encouraging activity without dose-limiting toxicities. As per protocol design, hydroxyurea (HU) or anagrelide as cytoreductive therapy was allowed, while prohibited medications included interferon, thalidomide, busulfan or lenalidomide, requiring a washout period of 5 half-live prior to study enrollment. A rapid reduction in platelet count was reported, with hematologic responses achieved within 4 weeks, an overall response rate (ORR) of 79%, and complete responses (CR) in 66% of patients. Reductions in variant allele frequency (VAF) were observed in 88% of patients, with 25% achieving a ≥50% reduction after 12 weeks of treatment. Most adverse events were grade 1–2, with transient, asymptomatic grade 3 events mainly consisting of lipase elevations [49]. Early dose-dependent normalization of platelet counts and reductions in clonal burden were observed; definitive results are awaited.

Additional antibodies are designed to recruit immune effector cells, mediating antibody-dependent cellular cytotoxicity (ADCC), antibody-dependent cellular phagocytosis (ADCP), and complement-dependent cytotoxicity (CDC). In 2023, Kuchnio et al. [50] reported JNJ-88549968, a bispecific T-cell–redirecting antibody selectively targeting MPN clones. This agent induced specific binding and T-cell activation against *CALR*-mutated cells in vitro, including autologous assays, and in xenograft models, regardless of mutation subtype; it is now in a phase 1 trial (NCT06150157).

Another T-cell–redirecting antibody, INCA035784, is a fully human IgG1 with a silenced Fc domain. Unlike INCA033989, it binds an N-domain region of mutant CALR conserved across C-terminal mutations [51]. It does not recognize wild-type CALR exposed on the cell surface after doxorubicin-induced stress, and binding is unaffected by soluble CALR. In vitro, it induces dose-dependent T-cell activation and cytotoxicity against CALR-mutated CD34+ cells from both type 1 and type 2 cases.

Finally, precision antibody–drug conjugates (pADCs) have been explored to deliver cytotoxic payloads selectively to malignant cells. One approach couples degraders of SMARCA2/4 or CDK9 to an anti-CALR MoAb, leveraging dysregulation of the SWI/SNF chromatin-remodeling complex, which governs gene expression programs essential for hematopoietic stem cell maintenance [52,53,54]. Dual degradation disrupts aberrant SWI/SNF function, inducing cytotoxicity in progenitors and restoring normal hematopoiesis. Preclinical data show selective binding to mutant cells, inhibition of megakaryocytic growth and differentiation in patient-derived *CALR*-mutated samples, and durable disease regression. Further studies are ongoing [52].

### 3.2. Vaccination Strategies Against Exon 9 CALR Mutations and Immune Checkpoint Inhibition

Therapeutic cancer vaccines aim to elicit immune responses directly against tumor-specific antigens [55]. In MPNs, exon 9 *CALR* mutations generate unique neoantigens, making them attractive targets. Early studies showed that patient-derived T cells could recognize and eliminate autologous CALR-mutated cells ex vivo, but their activity was weaker than that of healthy donor T cells stimulated with the same peptides [56,57]. To optimize immunogenicity, Gigoux et al. [58] used in silico prediction to identify mutant CALR neoepitopes with strong MHC-I binding; however, these alleles were underrepresented in *CALR*-mutated compared to *JAK2*-mutated cases, potentially restricting vaccine applicability.

Clinically, the CALRlong36 peptide vaccine was evaluated in the phase 1 trial NCT03566446 [59]. Ten *CALR*-mutated MPN patients received 15 doses over one year. Ex vivo T-cell responses emerged in 8 patients, yet no hematologic or molecular responses were achieved. Attempts to enhance efficacy through combinatorial approaches have so far been unsuccessful. In NCT05444530, dual CALR/JAK2 vaccination was combined with the CTLA-4 inhibitor ipilimumab in 14 patients [60]. Mutant CALR-specific immune responses were observed in 35.7%, but no reduction in allele burden occurred, leading to early trial discontinuation.

### 3.3. CAR T-Cell Therapy Targeting Mutant CALR

Preclinical efforts have also extended to CAR T-cell therapy. Schueller et al. [61] showed that murine CALR-mutant-specific CAR-T cells could target Ba/F3-hMPL cells expressing human CALR^Del52^ and prolong survival in xenografts, although no remissions were achieved in immunocompetent chimeric mice. More recently, Rampotas et al. [62] reported a CALR-directed CAR T-cell strategy capable of selectively eradicating CALR-mutated human cell lines, irrespective of expression levels. In NGS xenografts harboring *CALR*-mutated AML cells, this approach reduced leukemic burden and improved survival. In ex vivo assays using CD34+ cells from MPN patients, depletion ranged from 40% to 90% with limited off-target toxicity toward *JAK2* V617F samples.

## 4. Targeting CALR in the Era of Precision Medicine: Issues to Be Addressed

From the earliest observations it became clear that *CALR*-mutated MPNs display distinctive clinical features compared with other driver mutations. Indeed, *CALR*-mutated patients, as first reported by Klampfl and Nangalia [4,5], are younger and characteristically show marked thrombocytosis accompanied by relatively lower hemoglobin and leukocyte counts when compared with *JAK2*-mutated cases. In ET, *CALR* mutations further delineate a subgroup with reduced thrombotic risk [63]. While survival in ET appears independent of driver mutation status [64,65], type 1 *CALR* mutations in MF are consistently associated with improved outcomes [66,67,68]. These findings have informed the evolution of prognostic scoring systems [69]. It is important to note, however, that *CALR* attenuate but do not eliminate the adverse prognostic impact of high molecular risk (HMR) mutations and unfavorable karyotype [70]. These clinical and prognostic insights raise key considerations for the development of therapies targeting *CALR*-mutated clones.

### 4.1. Will Anti-CALR Therapies Shift Management from Thrombotic Risk Reduction to Disease Modification?

An open question is how anti-CALR therapies might reshape current management strategies and in which clinical settings their use would be most impactful. The potential to modify the natural history of the disease by directly targeting the mutant clone raises the issue of whether therapeutic goals should move beyond thrombotic risk reduction. This is especially relevant in ET, where current management is primarily focused on reducing thrombotic risk. While HU remains the standard treatment for high-risk IPSET-thrombosis patients and intermediate-risk cases requiring cytoreduction, unmet therapeutic needs persist. Anagrelide can be considered in patients who are resistant or intolerant to HU [71], although clinically significant anemia and long-term bone marrow fibrosis, particularly in *CALR*-mutated cases, have raised concerns [72,73]. Busulfan and pipobroman are effective alternatives but carry potential leukemogenic risks [74]. In addition, ruxolitinib did not demonstrate superiority over HU in the MAJIC-ET trial [75]. By contrast, pegylated interferon-α (Peg-IFNα) has shown efficacy in controlling thrombocytosis, reducing spleen size, and improving symptom burden [76,77], with notable molecular responses, particularly in *JAK2* V617F–mutated cases. Similar results have recently been reported for ropeginterferon α-2b (Ropeg-IFN) in patients with PV, and full results from ongoing studies in ET, including the SURPASS-ET trial, are eagerly awaited [78].

Similar issues arise in *CALR*-mutated MF, where management largely follows the principles applied to other driver mutations, yet distinct clinical considerations warrant attention. Pivotal trials of ruxolitinib and fedratinib have only partially addressed whether driver mutation status influences outcomes [79]. In a retrospective analysis of 29 CALR-mutated MF patients in the COMFORT-II trial, 20 received ruxolitinib and demonstrated safety, efficacy, and survival comparable to the overall cohort [80]. More recently, momelotinib was approved for MF with anemia due to its dual JAK1/JAK2 inhibition and blockade of activin A receptor type 1 (ACVR1). Among 79 JAK inhibitor-naïve patients treated with momelotinib, 14% carried type 1 CALR and 3% type 2 CALR mutations [81], with type 1 *CALR* patients showing significantly higher 3-, 5-, and 10-year survival even after multivariate adjustment for transplantation. Real-world data highlight additional nuances. In a cohort of 1.055 MF patients treated with ruxolitinib, 135 with *CALR* mutations more frequently developed anemia and increased blast percentages [82], likely reflecting later treatment initiation, higher disease burden, and longer intervals from diagnosis, which may diminish the favorable prognostic impact of *CALR* mutations.

Allogeneic hematopoietic stem cell transplantation (HSCT) remains the only curative option. Advances in technique and prognostic tools have broadened its feasibility. In 2023, Hernández-Boluda et al. [83] reported outcomes in 346 *CALR*-mutated MF patients undergoing HSCT, with five-year survival of 63% versus 50% in JAK2-mutated cases, though no distinction was made by *CALR* subtype or HMR status. More recently, Gagelmann et al. [84] analyzed clonal dynamics in 324 patients after reduced-intensity conditioning: 23% were *CALR*-mutated, achieving molecular clearance in 73% at day 30 and 82% at day 100, compared with 42% and 63% for JAK2-mutated cases and 54% and 100% for *MPL*-mutated cases, potentially explaining the superior post-transplant outcomes in *CALR*- and *MPL*-mutated patients.

Taken together, anti-CALR therapies represent an important opportunity to achieve disease modification, something rarely attainable with current approaches. As ongoing studies mature, they will clarify whether these agents can meaningfully reduce thrombotic risk, delay disease progression, and improve quality of life in patients with ET and MF.

### 4.2. Are We Ready for Measurable Residual Disease Monitoring?

The prospect of targeted therapy capable of eradicating the mutant clone highlights the importance of molecular monitoring, akin to measurable residual disease (MRD), to evaluate treatment efficacy over time and guide clinical decisions. Improvements in blood counts, symptoms, or organ involvement do not always correlate with molecular responses [85].

Although current management of PV and ET focuses largely on thrombo-hemorrhagic risk reduction, preventing fibrotic or leukemic progression remains a critical research goal. This is supported by evidence linking disease progression to increasing allele burden during follow-up [86]. While the *JAK2* V617F VAF exhibits considerable interindividual variability in MPNs, *CALR*-mutated cases show a more homogeneous distribution, typically between 40% and 50% [87,88,89]. In patients with *CALR* VAF ≤20%, lower platelet and neutrophil counts and improved overall survival have been reported, independent of mutation subtype, age, or thrombotic history [88]. Conversely, higher allele burdens are associated with increased acquisition of additional mutations, greater risk of anemia during follow-up, and more frequent progression to fibrosis [87,89].

To date, no therapy has convincingly altered the natural history of MPNs. As previously said, IFNα has demonstrated not only complete hematologic responses but also molecular responses, outcomes not achieved with cytoreduction using anagrelide or HU [90]. However, in *CALR*-mutated patients, clonal reduction dynamics are variable even among molecular responders [91], and the reduction of the *CALR*-mutant clone with Peg-IFNα is generally less pronounced than in *JAK2* V617F-mutated patients [92,93]. The MAJIC-PV trial provided evidence that molecular responses in PV patients treated with ruxolitinib correlate with improved overall and progression-free survival [94]. In addition, Guglielmelli et al. [85] prospectively measured changes in *JAK2* and *CALR* VAF in 77 PV or ET patients treated with ruxolitinib, reporting reductions from a median of 68% to 3.5% in *JAK2*-mutated cases and from 49% to 4% in a *CALR*-mutated ET patient. Molecular responses were also observed in three *CALR*-mutated ET patients treated with ruxolitinib in the MAJIC-ET study [75].

Several techniques have been evaluated for monitoring allele burden, including fragment length analysis (FLA), Sanger sequencing, and next-generation sequencing (NGS) [86,91]. Jones et al. [95] compared detection limits of *CALR* assays using cell lines with 61 bp deletions, reporting detection limits of 10–25%, 5%, 5%, and 1.2% for Sanger sequencing, FLA, high-resolution melt analysis, and NGS, respectively. While FLA is rapid and cost-effective for diagnostic screening, it has a relatively high detection limit and is unsuitable for precise quantification of fragments of different sizes, often overestimating shorter fragments [91,96]. Similarly, Sanger sequencing, with 10–20% sensitivity, is inadequate for MRD monitoring [97]. NGS is highly sensitive but limited by cost and turnaround time, particularly for allele burdens below 1–5%. Quantitative PCR (qPCR) offers higher sensitivity and broad availability, but its application to CALR is challenged by the complex assay design required due to nucleotide repeats in exon 9. This approach has been employed to quantify type 1 and type 2 mutations, with a detection limit that generally does not exceed 0.1%, remaining suboptimal for reliable MRD monitoring [97].

In contrast, digital droplet PCR (ddPCR) overcomes the limitations of conventional PCR by partitioning the reaction into thousands of microdroplets, each analyzed individually. The low DNA copy number per droplet minimizes competition between primers, enabling highly accurate quantification of allele burden [84,86,91]. Using this approach, mutant CALR alleles can be detected down to 0.01%, providing a highly sensitive and precise tool for MRD assessment [96,98]. A clear example of its clinical utility is the monitoring of MRD post-HSCT in myelofibrosis. Early studies demonstrated that *CALR* mutations are cleared more rapidly and frequently post-transplant compared with *MPL* or *JAK2* V617F mutations [99]. Patients with detectable mutations at days +100 and +180 had a significantly higher risk of relapse, irrespective of the underlying driver mutation. More recent evidence indicates that mutation clearance as early as day +30 is a strong predictor of both relapse and post-transplant survival, emphasizing the importance of timely molecular monitoring [84].

Nevertheless, defining molecular response as a marker of disease modification requires careful standardization and validation of threshold levels, which must ultimately translate into meaningful clinical benefit. Importantly, clearance of the dominant mutant clone does not necessarily equate to clearance of all clones, highlighting the need for further studies to clarify the clinical relevance of clonal monitoring in MPNs.

### 4.3. How to Face the Immune Escape Issue?

Accumulating evidence supports the presence of an immunosuppressive environment in CALR-mutated MPNs. While the underlying mechanisms remain incompletely understood, they appear primarily related to impaired antigen presentation and immunosuppressive remodeling of the tumor microenvironment. Impaired phagocytosis of tumor cells has been observed in CALR^Del52^ knock-in mice, consistent with an immunosuppressive role of CALR-mutated clones; this defect was associated with reduced T-cell activation and expansion of regulatory T cells (Tregs) [100], rather than affecting phagocytic capacity of macrophages [101]. Additional studies have shown that CALR^Del52^ variant drives the expansion of TGF-β1-producing erythroid progenitors and Tregs, while suppressing T-cell cytotoxicity [102].

Moreover, although CALR mutations can elicit T-cell responses, *CALR*-mutant-specific responses are attenuated in MPN patients compared with healthy individuals [103]. High expression of immune checkpoint receptors such as PD-1 and CTLA-4, consistent with T-cell exhaustion, has been reported. Furthermore, CALR-mutant–specific T cells from patients are predominantly CD4+, and the absence of CALR-mutant-specific CD8+ T cells may reflect impaired antigen presentation [103]. Under normal conditions, CALR participates in the peptide-loading complex (PLC) along with ERp57 and tapasin, ensuring proper assembly and high-affinity peptide loading onto major histocompatibility complex (MHC) class I molecules [104]. Proteasome-derived peptides are transported into the endoplasmic reticulum by the TAP1/TAP2 heterodimer, trimmed by ERAP1, and loaded onto MHC class I heavy chains and β2-microglobulin with the assistance of tapasin, ERp57, and CALR. Fully assembled complexes are then exported via the Golgi to the cell surface to mediate immune recognition of abnormal cells [105]. By guiding MHC class I molecules to the PLC and stabilizing peptide loading, CALR is essential for antigen presentation. Mutant CALR disrupts this process, impairs high-affinity peptide binding, and reduces MHC class I expression at the cell surface, likely facilitating immune evasion of CALR-mutated clones [106].

Collectively, these findings highlight immune escape as a major barrier in CALR-mutated MPNs, likely contributing to the limited success of T-cell-based and vaccination strategies. Continued efforts to overcome this hurdle will be essential for the development of effective therapies.

## 5. Conclusions

*CALR* mutations and their downstream pathophysiologic effects offer unique opportunities for precision targeting. Despite highly encouraging preclinical results demonstrating selectivity and low toxicity, challenges persist. While the biological rationale and preclinical evidence strongly support the development of mutant CALR-directed therapies, clinical success will require overcoming disease-intrinsic immune evasion, refining patient selection, and implementing robust strategies for clonal burden monitoring.

## Figures and Tables

**Table 1:** Ongoing Phase I clinical trials in MPN patients with documented *CALR* exon 9 mutation.

NCT Identifies	Drug	Population Study	Primary Endpoint
**Anti-CALR Monoclonal Antibodies**			
NCT05936359 NCT06034002	INCA033989	HR ^†^ ET, with platelets > 450 × 10^9^/L; resistant to or intolerant to ≥1 prior cytoreductive therapy (concomitant therapy with ANA or HU permitted) DIPSS IR/HR PMF/Post-ET MF previously treated with JAKi for >12 weeks; resistant/refractory/intolerant/loss response to JAKi; blast < 10%;	DLTs, TEAEs, and TEAEs ^‡^ leading to dose modification or discontinuation
NCT07008118	INCA035784	DIPSS+ IR-2/HR MF with prior JAKi, <20% blasts, and measurable spleen; resistant, refractory, intolerant, or has lost response to ≥1 prior line of therapy HR ET with platelets >450 × 10^9^/L and ≥2 prior treatment lines	DLTs, TEAEs, and TEAEs leading to treatment interruption, discontinuation or delay
NCT06150157	JNJ-88549968	*CALR*-mutated ET and MF	DLTs, AEs and AEs by severity
**Anti-CALR Vaccination**			
NCT05025488	Peptide-based vaccine and Poly-ICLC ^§^	HR ET; previously treated or relapsed/refractory LR/IR-1 (DIPSS 0-1) PMF or post-ET MF	DLTs
NCT03566446	CALRLong36 peptide	ET, post-ET MF, prefibrotic MF, overt MF	AEs
NCT05444530	VAC85135 + ipilimumab	*CALR*-mutated MPN (type 1/2 or like) or *JAK2* V617F with HLA-A*02:01	DLTs, AEs, serious AEs

^†^ Defined as age ≥60 years or history of thrombosis or history of major bleeding without any clearly documented alternative explanation or extreme thrombocytosis. ^‡^ Only for NCT06034002 at ClinicalTrials.gov. ^§^ Immunostimulatory adjuvant. Abbreviations: AEs, Adverse events; ANA, anagrelide; DIPSS, Dynamic International Prognostic Scoring System; DLTs, Dose Limiting Toxicities; ET, essential thrombocythemia; HR, high risk; HU, hydroxyurea; JAKi, JAK inhibitor; MF, myelofibrosis; TEAEs, Treatment-emergent Adverse Events.

## Data Availability

No new data were created or analyzed in this study.

## Abbreviations

The following abbreviations are used in this manuscript:

ACVR1	Activin A receptor type 1
ADCC	Antibody-dependent cellular cytotoxicity
ADCP	Antibody-dependent cellular phagocytosis
CAR-T	Chimeric antigen receptor T cell
CDC	Complement-dependent cytotoxicity
CR	Complete response
ET	Essential Thrombocytemia
ER	Endoplasmic reticulum
FLA	Fragment length analysis
HMR	High molecular risk
HSCT	Hematopoietic stem cell transplantation
HU	Hydroxyurea
MoAbs	Monoclonal antibodies
MPN	Myeloproliferative neoplasm
MHC MRD	Major histocompatibility complex Measurable residual disease
NGS	Next-generation sequencing
ORR	Overall response rate
pADCs	Precision antibody–drug conjugates
PCR	Polymerase chain reaction
PLC	Peptide-loading complex
PV	Polycythemia Vera
PMF	Primary Myelofibrosis
smCALR	Soluble mutant CALR
TPO	Thrombopoietin
Tregs	Regulatory T cells
VAF	Variant allele frequency

## References

[1] Arber D.A., Orazi A., Hasserjian R.P., Borowitz M.J., Calvo K.R., Kvasnicka H.-M., Wang S.A., Bagg A., Barbui T., Branford S. (2022). International Consensus Classification of Myeloid Neoplasms and Acute Leukemias: Integrating Morphologic, Clinical, and Genomic Data. Blood.

[2] Adamson J.W., Fialkow P.J., Murphy S., Prchal J.F., Steinmann L. (1976). Polycythemia Vera: Stem-Cell and Probable Clonal Origin of the Disease. N. Engl. J. Med..

[3] Pikman Y., Lee B.H., Mercher T., McDowell E., Ebert B.L., Gozo M., Cuker A., Wernig G., Moore S., Galinsky I. (2006). MPLW515L Is a Novel Somatic Activating Mutation in Myelofibrosis with Myeloid Metaplasia. PLoS Med..

[4] Nangalia J., Massie C.E., Baxter E.J., Nice F.L., Gundem G., Wedge D.C., Avezov E., Li J., Kollmann K., Kent D.G. (2013). Somatic CALR Mutations in Myeloproliferative Neoplasms with Nonmutated JAK2. N. Engl. J. Med..

[5] Klampfl T., Gisslinger H., Harutyunyan A.S., Nivarthi H., Rumi E., Milosevic J.D., Them N.C.C., Berg T., Gisslinger B., Pietra D. (2013). Somatic Mutations of Calreticulin in Myeloproliferative Neoplasms. N. Engl. J. Med..

[6] Michalak M., Groenendyk J., Szabo E., Gold L.I., Opas M. (2009). Calreticulin, a Multi-Process Calcium-Buffering Chaperone of the Endoplasmic Reticulum. Biochem. J..

[7] Houen G., Højrup P., Ciplys E., Gaboriaud C., Slibinskas R., Agellon L.B., Michalak M. (2021). Structural Analysis of Calreticulin, an Endoplasmic Reticulum-Resident Molecular Chaperone. Cellular Biology of the Endoplasmic Reticulum.

[8] Wijeyesakere S.J., Gafni A.A., Raghavan M. (2011). Calreticulin Is a Thermostable Protein with Distinct Structural Responses to Different Divalent Cation Environments. J. Biol. Chem..

[9] Peterson J.R., Ora A., Van P.N., Helenius A. (1995). Transient, Lectin-Like Association of Calreticulin with Folding Intermediates of Cellular and Viral Glycoproteins. Mol. Biol. Cell.

[10] Schürch P.M., Malinovska L., Hleihil M., Losa M., Hofstetter M.C., Wildschut M.H.E., Lysenko V., Lakkaraju A.K.K., Maat C.A., Benke D. (2022). Calreticulin Mutations Affect Its Chaperone Function and Perturb the Glycoproteome. Cell Rep..

[11] Obeid M., Tesniere A., Ghiringhelli F., Fimia G.M., Apetoh L., Perfettini J.L., Castedo M., Mignot G., Panaretakis T., Casares N. (2007). Calreticulin exposure dictates the immunogenicity of cancer cell death. Nat. Med..

[12] Wijeyesakere S.J., Bedi S.K., Huynh D., Raghavan M. (2016). The C-terminal acidic region of calreticulin mediates phosphatidylserine binding and apoptotic cell phagocytosis. J. Immunol..

[13] Feng M., Chen J.Y., Weissman-Tsukamoto R., Volkmer J.P., Ho P.Y., McKenna K.M., Cheshier S., Zhang M., Guo N., Gip P. (2015). Macrophages eat cancer cells using their own calreticulin as a guide: Roles of TLR and Btk. Proc. Natl. Acad. Sci. USA.

[14] Tefferi A., Lasho T.L., Tischer A., Wassie E.A., Finke C.M., Belachew A.A., Ketterling R.P., Hanson C.A., Pardanani A.D. (2014). The Prognostic Advantage of Calreticulin Mutations in Myelofibrosis Might Be Confined to Type 1 or Type 1-like CALR Variants. Blood.

[15] Li B., Xu Z., Li Y., Peter Gale R., Song Z., Ai X., Qin T., Zhang Y., Zhang P., Huang G. (2016). The Different Prognostic Impact of Type-1 or Type-1 like and Type-2 or Type-2 like CALR Mutations in Patients with Primary Myelofibrosis. Am. J. Hematol..

[16] Guglielmelli P., Rotunno G., Fanelli T., Pacilli A., Brogi G., Calabresi L., Pancrazzi A., Vannucchi A.M. (2015). Validation of the Differential Prognostic Impact of Type 1/Type 1-like versus Type 2/Type 2-like CALR Mutations in Myelofibrosis. Blood Cancer J..

[17] Elf S., Abdelfattah N.S., Chen E., Perales-Patón J., Rosen E.A., Ko A., Peisker F., Florescu N., Giannini S., Wolach O. (2016). Mutant Calreticulin Requires Both Its Mutant C-Terminus and the Thrombopoietin Receptor for Oncogenic Transformation. Cancer Discov..

[18] Nivarthi H., Chen D., Cleary C., Kubesova B., Jäger R., Bogner E., Marty C., Pecquet C., Vainchenker W., Constantinescu S.N. (2016). Thrombopoietin Receptor Is Required for the Oncogenic Function of CALR Mutants. Leukemia.

[19] Chachoua I., Pecquet C., El-Khoury M., Nivarthi H., Albu R.-I., Marty C., Gryshkova V., Defour J.-P., Vertenoeil G., Ngo A. (2016). Thrombopoietin Receptor Activation by Myeloproliferative Neoplasm Associated Calreticulin Mutants. Blood.

[20] Araki M., Yang Y., Masubuchi N., Hironaka Y., Takei H., Morishita S., Mizukami Y., Kan S., Shirane S., Edahiro Y. (2016). Activation of the Thrombopoietin Receptor by Mutant Calreticulin in CALR-Mutant Myeloproliferative Neoplasms. Blood.

[21] Elf S., Abdelfattah N.S., Baral A.J., Beeson D., Rivera J.F., Ko A., Florescu N., Birrane G., Chen E., Mullally A. (2018). Defining the Requirements for the Pathogenic Interaction between Mutant Calreticulin and MPL in MPN. Blood.

[22] Salati S., Genovese E., Carretta C., Zini R., Bartalucci N., Prudente Z., Pennucci V., Ruberti S., Rossi C., Rontauroli S. (2019). Calreticulin Ins5 and Del52 Mutations Impair Unfolded Protein and Oxidative Stress Responses in K562 Cells Expressing CALR Mutants. Sci. Rep..

[23] Nam A.S., Kim K.-T., Chaligne R., Izzo F., Ang C., Taylor J., Myers R.M., Abu-Zeinah G., Brand R., Omans N.D. (2019). Somatic Mutations and Cell Identity Linked by Genotyping of Transcriptomes. Nature.

[24] Jutzi J.S., Marneth A.E., Jiménez-Santos M.J., Hem J., Guerra-Moreno A., Rolles B., Bhatt S., Myers S.A., Carr S.A., Hong Y. (2023). CALR-Mutated Cells Are Vulnerable to Combined Inhibition of the Proteasome and the Endoplasmic Reticulum Stress Response. Leukemia.

[25] Papadopoulos N., Nédélec A., Derenne A., Şulea T.A., Pecquet C., Chachoua I., Vertenoeil G., Tilmant T., Petrescu A.-J., Mazzucchelli G. (2023). Oncogenic CALR Mutant C-Terminus Mediates Dual Binding to the Thrombopoietin Receptor Triggering Complex Dimerization and Activation. Nat. Commun..

[26] Pecquet C., Chachoua I., Roy A., Balligand T., Vertenoeil G., Leroy E., Albu R.-I., Defour J.-P., Nivarthi H., Hug E. (2019). Calreticulin Mutants as Oncogenic Rogue Chaperones for TpoR and Traffic-Defective Pathogenic TpoR Mutants. Blood.

[27] Araki M., Yang Y., Imai M., Mizukami Y., Kihara Y., Sunami Y., Masubuchi N., Edahiro Y., Hironaka Y., Osaga S. (2019). Homomultimerization of Mutant Calreticulin Is a Prerequisite for MPL Binding and Activation. Leukemia.

[28] Pecquet C., Papadopoulos N., Balligand T., Chachoua I., Tisserand A., Vertenoeil G., Nédélec A., Vertommen D., Roy A., Marty C. (2023). Secreted Mutant Calreticulins as Rogue Cytokines in Myeloproliferative Neoplasms. Blood.

[29] Hormoz S., Sankaran V.G., Mullally A. (2025). Evolution of Myeloproliferative Neoplasms from Normal Blood Stem Cells. Haematologica.

[30] Van Egeren D., Escabi J., Nguyen M., Liu S., Reilly C.R., Patel S., Kamaz B., Kalyva M., DeAngelo D.J., Galinsky I. (2021). Reconstructing the Lineage Histories and Differentiation Trajectories of Individual Cancer Cells in Myeloproliferative Neoplasms. Cell Stem Cell.

[31] Williams N., Lee J., Mitchell E., Moore L., Baxter E.J., Hewinson J., Dawson K.J., Menzies A., Godfrey A.L., Green A.R. (2022). Life Histories of Myeloproliferative Neoplasms Inferred from Phylogenies. Nature.

[32] Sousos N., Leathlobhair M.N., Karali C.S., Louka E., Bienz N., Royston D., Clark S.-A., Hamblin A., Howard K., Mathews V. (2022). In utero origin of myelofibrosis presenting in adult monozygotic twins. Nat. Med..

[33] Hermange G., Rakotonirainy A., Bentriou M., Tisserand A., El-Khoury M., Girodon F., Marzac C., Vainchenker W., Plo I., Cournède P.-H. (2022). Inferring the Initiation and Development of Myeloproliferative Neoplasms. Proc. Natl. Acad. Sci. USA.

[34] Olschok K., Han L., de Toledo M.A.S., Böhnke J., Graßhoff M., Costa I.G., Theocharides A., Maurer A., Schüler H.M., Buhl E.M. (2021). CALR frameshift mutations in MPN patient-derived iPSCs accelerate maturation of megakaryocytes. Stem Cell Rep..

[35] Foßelteder J., Pabst G., Sconocchia T., Schlacher A., Auinger L., Kashofer K., Beham-Schmid C., Trajanoski S., Waskow C., Schöll W. (2023). Human Gene-Engineered Calreticulin Mutant Stem Cells Recapitulate MPN Hallmarks and Identify Targetable Vulnerabilities. Leukemia.

[36] Li J., Prins D., Park H.J., Grinfeld J., Gonzalez-Arias C., Loughran S., Dovey O.M., Klampfl T., Bennett C., Hamilton T.L. (2018). Mutant Calreticulin Knockin Mice Develop Thrombocytosis and Myelofibrosis without a Stem Cell Self-Renewal Advantage. Blood.

[37] Shide K., Kameda T., Yamaji T., Sekine M., Inada N., Kamiunten A., Akizuki K., Nakamura K., Hidaka T., Kubuki Y. (2017). Calreticulin Mutant Mice Develop Essential Thrombocythemia That Is Ameliorated by the JAK Inhibitor Ruxolitinib. Leukemia.

[38] Achyutuni S., Nivarthi H., Majoros A., Hug E., Schueller C., Jia R., Varga C., Schuster M., Senekowitsch M., Tsiantoulas D. (2021). Hematopoietic Expression of a Chimeric Murine-Human CALR Oncoprotein Allows the Assessment of Anti-CALR Antibody Immunotherapies in Vivo. Am. J. Hematol..

[39] Benlabiod C., Cacemiro M.d.C., Nédélec A., Edmond V., Muller D., Rameau P., Touchard L., Gonin P., Constantinescu S.N., Raslova H. (2020). Calreticulin Del52 and Ins5 Knock-in Mice Recapitulate Different Myeloproliferative Phenotypes Observed in Patients with MPN. Nat. Commun..

[40] Shide K., Kameda T., Kamiunten A., Ozono Y., Tahira Y., Yokomizo-Nakano T., Kubota S., Ono M., Ikeda K., Sekine M. (2020). Calreticulin Haploinsufficiency Augments Stem Cell Activity and Is Required for Onset of Myeloproliferative Neoplasms in Mice. Blood.

[41] Shide K., Kameda T., Kamiunten A., Oji A., Ozono Y., Sekine M., Honda A., Kitanaka A., Akizuki K., Tahira Y. (2019). Mice with Calr Mutations Homologous to Human CALR Mutations Only Exhibit Mild Thrombocytosis. Blood Cancer J..

[42] Balligand T., Achouri Y., Pecquet C., Gaudray G., Colau D., Hug E., Rahmani Y., Stroobant V., Plo I., Vainchenker W. (2020). Knock-in of Murine Calr Del52 Induces Essential Thrombocythemia with Slow-Rising Dominance in Mice and Reveals Key Role of Calr Exon 9 in Cardiac Development. Leukemia.

[43] Tang L., Huang Z., Mei H., Hu Y. (2023). Immunotherapy in Hematologic Malignancies: Achievements, Challenges and Future Prospects. Signal Transduct. Target. Ther..

[44] Kihara Y., Araki M., Imai M., Mori Y., Horino M., Ogata S., Yoshikawa S., Taguchi T., Masubuchi N., Mabuchi Y. (2020). Therapeutic Potential of an Antibody Targeting the Cleaved Form of Mutant Calreticulin in Myeloproliferative Neoplasms. Blood.

[45] Mughal F.P., Bergmann A.C., Huynh H.U.B., Jørgensen S.H., Mansha I., Kesmez M., Schürch P.M., Theocharides A.P.A., Hansen P.R., Friis T. (2022). Production and Characterization of Peptide Antibodies to the C-Terminal of Frameshifted Calreticulin Associated with Myeloproliferative Diseases. Int. J. Mol. Sci..

[46] Tvorogov D., Thompson-Peach C.A., Foßelteder J., Dottore M., Stomski F., Onnesha S.A., Lim K., Moretti P.A., Pitson S.M., Ross D.M. (2022). Targeting Human CALR-mutated MPN Progenitors with a Neoepitope-directed Monoclonal Antibody. EMBO Rep..

[47] Reis E.S., Buonpane R., Celik H., Marty C., Lei A., Jobe F., Rupar M., Zhang Y., DiMatteo D., Awdew R. (2024). Selective Targeting of Mutated Calreticulin by the Monoclonal Antibody INCA033989 Inhibits Oncogenic Function of MPN. Blood.

[48] Marty C., Rosa E., Evrard M., Pegliasco J., Chedeville A., Blampey Q., Aid Z., Mercher T., Legros L., Fouquet G. (2024). Efficacy of INCA033989 in Chronic and Advanced Forms of CALRdel52 and CALRins5 Myeloproliferative Neoplasms (MPN) Models. HemaSphere.

[49] Mascarenhas J., Ali H., Yacoub A., Jain T., Chee L., Gupta V., Harrison C., Kiladjian J.-J., Mesa R., Shomali W. INCA33989 Is a Novel, First in Class, Mutant Calreticulin-Specific Monoclonal Antibody That Demonstrates Safety and Efficacy in Patients with Essential Thrombocythemia. Presented at the Annual Meeting of the European Hematology Association.

[50] Kuchnio A., Samakai E., Hug E., Balmaña M., Janssen L., Amorim R., Cornelissen I., Majoros A., Broux M., Taneja I. (2023). Discovery of JNJ-88549968, a Novel, First-in-Class CALRmutxCD3 T-Cell Redirecting Antibody for the Treatment of Myeloproliferative Neoplasms. Blood.

[51] Pandey V., Wang L.-C., Kulkarni A., Hendriks L., Steevels T., Merenich D., Guan J., Ren E., Langalia N., Fiedorczuk K. (2025). INCA035784, a novel, equipotent T cell-redirecting antibody for patients with myeloproliferative neoplasms carrying different types of calreticulin mutations. HemaSphere.

[52] Fultang N., Schwab A., Chandratre S., Johnson I., Karwoski J., Bhagwat N., Zou Y., Buesking A., Foroutan M., Chen C. (2025). Discovery of First-in-Class Precision Antibody Drug Conjugates Targeting Mutant Calreticulin for the Treatment of Myeloproliferative Neoplasms. HemaSphere.

[53] Fultang N., Schwab A.M., Johnson I., Grego A., Filler E., Bachner C., Karwoski J., Moore A., Bartilomo A., Agarwal A. (2024). Selective SMARCA2 Degradation Promotes Leukemic Differentiation and Synergizes with CDK9 Inhibition to Potently Induce Death in Pre-Clinical Models of Acute Myeloid Leukemia. Blood.

[54] Cantley J., Ye X., Rousseau E., Januario T., Hamman B.D., Rose C.M., Cheung T.K., Hinkle T., Soto L., Quinn C. (2022). Selective PROTAC-Mediated Degradation of SMARCA2 Is Efficacious in SMARCA4 Mutant Cancers. Nat. Commun..

[55] Zaidi N., Jaffee E.M., Yarchoan M. (2025). Recent Advances in Therapeutic Cancer Vaccines. Nat. Rev. Cancer.

[56] Holmström M.O., Riley C.H., Svane I.M., Hasselbalch H.C., Andersen M.H. (2016). The CALR Exon 9 Mutations Are Shared Neoantigens in Patients with CALR Mutant Chronic Myeloproliferative Neoplasms. Leukemia.

[57] Holmström M.O., Ahmad S.M., Klausen U., Bendtsen S.K., Martinenaite E., Riley C.H., Svane I.M., Kjær L., Skov V., Ellervik C. (2019). High Frequencies of Circulating Memory T Cells Specific for Calreticulin Exon 9 Mutations in Healthy Individuals. Blood Cancer J..

[58] Gigoux M., Holmström M.O., Zappasodi R., Park J.J., Pourpe S., Bozkus C.C., Mangarin L.M.B., Redmond D., Verma S., Schad S. (2022). Calreticulin Mutant Myeloproliferative Neoplasms Induce MHC-I Skewing, Which Can Be Overcome by an Optimized Peptide Cancer Vaccine. Sci. Transl. Med..

[59] Handlos Grauslund J., Holmström M.O., Jørgensen N.G., Klausen U., Weis-Banke S.E., El Fassi D., Schöllkopf C., Clausen M.B., Gjerdrum L.M.R., Breinholt M.F. (2021). Therapeutic Cancer Vaccination With a Peptide Derived From the Calreticulin Exon 9 Mutations Induces Strong Cellular Immune Responses in Patients With CALR-Mutant Chronic Myeloproliferative Neoplasms. Front. Oncol..

[60] Otoukesh S., Psaila B., Kuykendall A., Gerds A.T., Abdulgawad A., Bose P., Van Bogaert C., Bishop J., Wilkinson P., Liu B. A phase 1 study of VAC85135, a neoantigen vaccine regimen targeting calreticulin and JAK2 mutations, in combination with ipilimumab (IPI) in patients (PTS) with myeloproliferative neoplasms (MPNS). Presented at the Annual Meeting of the European Hematology Association.

[61] Schueller C., Varga C., Wais T., Höhrhan M., Xiong S., Balmanya M., Zagrijtschuk O., Kralovics R. Targeted T Cells against Hematopoietic Cells Expressing Oncogenic Calreticulin Mutants. Proceedings of the Annual Meeting of European Hematology Association.

[62] Rampotas A., Wong Z., Gannon I., Benlabiod C., Shen Y., Brierley C., Olijnik A.-A., Khan S., Hayder N., Cheung G.W.-K. (2024). Development of a First-in-Class CAR-T Therapy Against Calreticulin-Mutant Neoplasms and Evaluation in the Relevant Human Tissue Environment. Blood.

[63] Rotunno G., Mannarelli C., Guglielmelli P., Pacilli A., Pancrazzi A., Pieri L., Fanelli T., Bosi A., Vannucchi A.M., Associazione Italiana per la Ricerca sul Cancro Gruppo Italiano Malattie Mieloproliferative Investigators (2014). Impact of calreticulin mutations on clinical and hematological phenotype and outcome in essential thrombocythemia. Blood.

[64] Gangat N., Karrar O., Al-Kali A., Begna K.H., Elliott M.A., Wolanskyj-Spinner A.P., Pardanani A., Hanson C.A., Ketterling R.P., Tefferi A. (2024). One Thousand Patients with Essential Thrombocythemia: The Mayo Clinic Experience. Blood Cancer J..

[65] Loscocco G.G., Gesullo F., Capecchi G., Atanasio A., Maccari C., Mannelli F., Vannucchi A.M., Guglielmelli P. (2024). One Thousand Patients with Essential Thrombocythemia: The Florence-CRIMM Experience. Blood Cancer J..

[66] Tefferi A., Nicolosi M., Mudireddy M., Szuber N., Finke C.M., Lasho T.L., Hanson C.A., Ketterling R.P., Pardanani A., Gangat N. (2018). Driver Mutations and Prognosis in Primary Myelofibrosis: Mayo-Careggi MPN Alliance Study of 1095 Patients. Am. J. Hematol..

[67] Passamonti F., Giorgino T., Mora B., Guglielmelli P., Rumi E., Maffioli M., Rambaldi A., Caramella M., Komrokji R., Gotlib J. (2017). A Clinical-Molecular Prognostic Model to Predict Survival in Patients with Post Polycythemia Vera and Post Essential Thrombocythemia Myelofibrosis. Leukemia.

[68] Tefferi A., Lasho T.L., Finke C.M., Knudson R.A., Ketterling R., Hanson C.H., Maffioli M., Caramazza D., Passamonti F., Pardanani A. (2014). CALR vs JAK2 vs MPL-Mutated or Triple-Negative Myelofibrosis: Clinical, Cytogenetic and Molecular Comparisons. Leukemia.

[69] Mora B., Bucelli C., Cattaneo D., Bellani V., Versino F., Barbullushi K., Fracchiolla N., Iurlo A., Passamonti F. (2024). Prognostic and Predictive Models in Myelofibrosis. Curr. Hematol. Malig. Rep..

[70] Szuber N., Lasho T.L., Finke C., Hanson C.A., Ketterling R.P., Pardanani A., Gangat N., Tefferi A. (2019). Determinants of Long-Term Outcome in Type 1 Calreticulin-Mutated Myelofibrosis. Leukemia.

[71] Alvarez-Larrán A., Sant’Antonio E., Harrison C., Kiladjian J.J., Griesshammer M., Mesa R., Ianotto J.C., Palandri F., Hernández-Boluda J.C., Birgegård G. (2021). Unmet Clinical Needs in the Management of CALR-Mutated Essential Thrombocythaemia: A Consensus-Based Proposal from the European LeukemiaNet. Lancet Haematol..

[72] Bieniaszewska M., Sobieralski P., Leszczyńska A., Dutka M. (2022). Anagrelide in essential thrombocythemia: Efficacy and long-term consequences in young patient population. Leuk. Res..

[73] Mazzucconi M.G., Baldacci E., Latagliata R., Breccia M., Paoloni F., Di Veroli A., Cedrone M., Anaclerico B., Villivà N., Porrini R. (2020). Anagrelide in Essential Thrombocythemia (ET): Results from 150 Patients over 25 Years by the “Ph1-Negative Myeloproliferative Neoplasms Latium Group”. Eur. J. Haematol..

[74] Tefferi A., Vannucchi A.M., Barbui T. (2024). Essential Thrombocythemia: 2024 Update on Diagnosis, Risk Stratification, and Management. Am. J. Hematol..

[75] Harrison C.N., Mead A.J., Panchal A., Fox S., Yap C., Gbandi E., Houlton A., Alimam S., Ewing J., Wood M. (2017). Ruxolitinib vs Best Available Therapy for ET Intolerant or Resistant to Hydroxycarbamide. Blood.

[76] Quintás-Cardama A., Kantarjian H., Manshouri T., Luthra R., Estrov Z., Pierce S., Richie M.A., Borthakur G., Konopleva M., Cortes J. (2009). Pegylated Interferon Alfa-2a Yields High Rates of Hematologic and Molecular Response in Patients with Advanced Essential Thrombocythemia and Polycythemia Vera. J. Clin. Oncol..

[77] Kiladjian J.-J., Cassinat B., Chevret S., Turlure P., Cambier N., Roussel M., Bellucci S., Grandchamp B., Chomienne C., Fenaux P. (2008). Pegylated Interferon-Alfa-2a Induces Complete Hematologic and Molecular Responses with Low Toxicity in Polycythemia Vera. Blood.

[78] Mesa R.A., Gill H., Xiao Z., Komatsu N., Qin A., Tashi T., Zhang L., Jin J., Kirito K., Ohishi K. (2025). Ropeginterferon Alfa-2b versus Anagrelide for the Treatment of Essential Thrombocythemia: Topline Results of the Phase 3 SURPASS-ET Trial. J. Clin. Oncol..

[79] Passamonti F., Caramazza D., Maffioli M. (2014). JAK Inhibitor in CALR-Mutant Myelofibrosis. N. Engl. J. Med..

[80] Guglielmelli P., Rotunno G., Bogani C., Mannarelli C., Giunti L., Provenzano A., Giglio S., Squires M., Stalbovskaya V., Gopalakrishna P. (2016). Ruxolitinib Is an Effective Treatment for CALR-Positive Patients with Myelofibrosis. Br. J. Haematol..

[81] Tefferi A., Pardanani A., Begna K.H., Al-Kali A., Hogan W.J., Litzow M.R., Ketterling R.P., Reichard K.K., Gangat N. (2024). Calr Type 1/like Mutation in Myelofibrosis Is the Most Prominent Predictor of Momelotinib Drug Survival and Longevity without Transplant. Blood Cancer J..

[82] Palandri F., Branzanti F., Morsia E., Dedola A., Benevolo G., Tiribelli M., Beggiato E., Farina M., Martino B., Caocci G. (2025). Impact of Calreticulin Mutations on Treatment and Survival Outcomes in Myelofibrosis during Ruxolitinib Therapy. Ann. Hematol..

[83] Hernandez-Boluda J.-C., Pereira A., Zinger N., Gras L., Martino R., Paneesha S., Finke J., Chinea A., Rambaldi A., Robin M. (2022). Allogeneic Hematopoietic Cell Transplantation in Patients with Myeloid/Lymphoid Neoplasm with FGFR1-Rearrangement: A Study of the Chronic Malignancies Working Party of EBMT. Bone Marrow Transpl..

[84] Gagelmann N., Quarder M., Badbaran A., Rathje K., Janson D., Lück C., Richter J., Marquard F., Oechsler S., Massoud R. (2025). Clearance of Driver Mutations after Transplantation for Myelofibrosis. N. Engl. J. Med..

[85] Guglielmelli P., Mora B., Gesullo F., Mannelli F., Loscocco G.G., Signori L., Pessina C., Colugnat I., Aquila R., Balliu M. (2024). Clinical Impact of Mutated JAK2 Allele Burden Reduction in Polycythemia Vera and Essential Thrombocythemia. Am. J. Hematol..

[86] Cottin L., Riou J., Orvain C., Ianotto J.C., Boyer F., Renard M., Truchan-Graczyk M., Murati A., Jouanneau-Courville R., Allangba O. (2020). Sequential Mutational Evaluation of CALR -Mutated Myeloproliferative Neoplasms with Thrombocytosis Reveals an Association between CALR Allele Burden Evolution and Disease Progression. Br. J. Haematol..

[87] Guglielmelli P., Szuber N., Gangat N., Capecchi G., Maccari C., Harnois M., Karrar O., Abdelmagid M., Balliu M., Nacca E. (2024). CALR Mutation Burden in Essential Thrombocythemia and Disease Outcome. Blood.

[88] Aubin L., Vilas Boas R., Daltro De Oliveira R., Le Brun V., Divoux M., Rey J., Mansier O., Ianotto J.-C., Pastoret C., Desmares A. (2024). CALR-Mutated Patients with Low Allele Burden Represent a Specific Subtype of Essential Thrombocythemia: A Study on Behalf of FIM and GBMHM. Am. J. Hematol..

[89] Mroczkowska-Bękarciak A., Szeremet A., Chyrko O., Wróbel T. (2025). CALR—Mutant Myeloproliferative Neoplasms: Insights from next-Generation Sequencing. J. Appl. Genet..

[90] Bewersdorf J.P., Giri S., Wang R., Podoltsev N., Williams R.T., Tallman M.S., Rampal R.K., Zeidan A.M., Stahl M. (2021). Interferon Alpha Therapy in Essential Thrombocythemia and Polycythemia Vera—A Systematic Review and Meta-Analysis. Leukemia.

[91] Kjær L., Cordua S., Holmström M.O., Thomassen M., Kruse T.A., Pallisgaard N., Larsen T.S., de Stricker K., Skov V., Hasselbalch H.C. (2016). Differential Dynamics of CALR Mutant Allele Burden in Myeloproliferative Neoplasms during Interferon Alfa Treatment. PLoS ONE.

[92] Czech J., Cordua S., Weinbergerova B., Baumeister J., Crepcia A., Han L., Maié T., Costa I.G., Denecke B., Maurer A. (2019). JAK2V617F but Not CALR Mutations Confer Increased Molecular Responses to Interferon-α via JAK1/STAT1 Activation. Leukemia.

[93] Stegelmann F., Teichmann L.L., Heidel F.H., Crodel C.C., Ernst T., Kreil S., Reiter A., Otten S., Schauer S., Körber R.-M. (2023). Clinicohematologic and Molecular Response of Essential Thrombocythemia Patients Treated with Pegylated Interferon-α: A Multi-Center Study of the German Study Group-Myeloproliferative Neoplasms (GSG-MPN). Leukemia.

[94] Harrison C.N., Nangalia J., Boucher R., Jackson A., Yap C., O’Sullivan J., Fox S., Ailts I., Dueck A.C., Geyer H.L. (2023). Ruxolitinib Versus Best Available Therapy for Polycythemia Vera Intolerant or Resistant to Hydroxycarbamide in a Randomized Trial. J. Clin. Oncol..

[95] Jones A.V., Ward D., Lyon M., Leung W., Callaway A., Chase A., Dent C.L., White H.E., Drexler H.G., Nangalia J. (2015). Evaluation of Methods to Detect CALR Mutations in Myeloproliferative Neoplasms. Leuk. Res..

[96] Mansier O., Migeon M., Saint-Lézer A., James C., Verger E., Robin M., Socié G., Bidet A., Mahon F.X., Cassinat B. (2016). Quantification of the Mutant CALR Allelic Burden by Digital PCR: Application to Minimal Residual Disease Evaluation after Bone Marrow Transplantation. J. Mol. Diagn..

[97] Chi J., Manoloukos M., Pierides C., Nicolaidou V., Nicolaou K., Kleopa M., Vassiliou G., Costeas P. (2015). Calreticulin Mutations in Myeloproliferative Neoplasms and New Methodology for Their Detection and Monitoring. Ann. Hematol..

[98] Anelli L., Zagaria A., Coccaro N., Tota G., Minervini A., Casieri P., Impera L., Minervini C.F., Brunetti C., Ricco A. (2016). Droplet digital PCR assay for quantifying CALR mutant allelic burden in myeloproliferative neoplasms. Ann. Hematol..

[99] Wolschke C., Badbaran A., Zabelina T., Christopeit M., Ayuk F., Triviai I., Zander A., Alchalby H., Bacher U., Fehse B. (2017). Impact of molecular residual disease post allografting in myelofibrosis patients. Bone Marrow Transpl..

[100] Liu P., Zhao L., Loos F., Marty C., Xie W., Martins I., Lachkar S., Qu B., Waeckel-Énée E., Plo I. (2020). Immunosuppression by Mutated Calreticulin Released from Malignant Cells. Mol. Cell.

[101] Daitoku S., Takenaka K., Yamauchi T., Yurino A., Jinnouchi F., Nunomura T., Eto T., Kamimura T., Higuchi M., Harada N. (2016). Calreticulin mutation does not contribute to disease progression in essential thrombocythemia by inhibiting phagocytosis. Exp. Hematol..

[102] Schmidt D., Endres C., Hoefflin R., Andrieux G., Zwick M., Karantzelis N., Staehle H.F., Vinnakota J.M., Duquesne S., Mozaffari Jovein M. (2024). Oncogenic Calreticulin Induces Immune Escape by Stimulating TGFβ Expression and Regulatory T-Cell Expansion in the Bone Marrow Microenvironment. Cancer Res..

[103] Bozkus C.C., Roudko V., Finnigan J.P., Mascarenhas J., Hoffman R., Iancu-Rubin C., Bhardwaj N. (2019). Immune Checkpoint Blockade Enhances Shared Neoantigen-Induced T Cell Immunity Directed against Mutated Calreticulin in Myeloproliferative Neoplasms. Cancer Discov..

[104] Wijeyesakere S.J., Gagnon J.K., Arora K., Brooks C.L., Raghavan M. (2015). Regulation of Calreticulin–Major Histocompatibility Complex (MHC) Class I Interactions by ATP. Proc. Natl. Acad. Sci. USA.

[105] Brunnberg J., Barends M., Frühschulz S., Winter C., Battin C., de Wet B., Cole D.K., Steinberger P., Tampé R. (2024). Dual Role of the Peptide-Loading Complex as Proofreader and Limiter of MHC-I Presentation. Proc. Natl. Acad. Sci. USA.

[106] Desikan H., Kaur A., Pogozheva I.D., Raghavan M. (2023). Effects of Calreticulin Mutations on Cell Transformation and Immunity. J. Cell. Mol. Med..

